# Supplementary material for: Anticancer Drug Response Prediction in Cell Lines Using Weighted Graph Regularized Matrix Factorization
Source: Mol Ther Nucleic Acids. 2019 Jun 4;17:164–74. doi: 10.1016/j.omtn.2019.05.017 (PMC6610642; doi:10.1016/j.omtn.2019.05.017)
Supplement: Document S1. Figures S1 and S2 and Table S1 [file mmc1.pdf]

**OMTN, Volume *17***

## Supplemental Information

# Anticancer Drug Response Prediction in Cell Lines Using Weighted Graph Regularized Matrix Factorization

**Na-Na Guan, Yan Zhao, Chun-Chun Wang, Jian-Qiang Li, Xing Chen, and Xue Piao**

# Anticancer drug response prediction in cell lines using weighted graph regularized matrix factorization

Na-Na Guan[1], Yan Zhao[2], Chun-Chun Wang[2], Jian-Qiang Li[1,*], Xing Chen[2,*], Xue Piao[3,*]

[1]College of Computer Science and Software Engineering, Shenzhen University, Shenzhen, 518060, China

[2]School of Information and Control Engineering, China University of Mining and Technology, Xuzhou, 221116, China

[3]School of Medical Informatics, Xuzhou Medical University, Xuzhou, 221004, China

*Corresponding author

**Email**: px@xzhmu.edu.cn; xingchen@amss.ac.cn; lijq@szu.edu.cn

**Keywords**: drug response; cell line; graph regularization; matrix factorization; response prediction

## Supplementary Information

**Supplementary Figure 1.** The distribution of known responses for all drugs in GDSC.

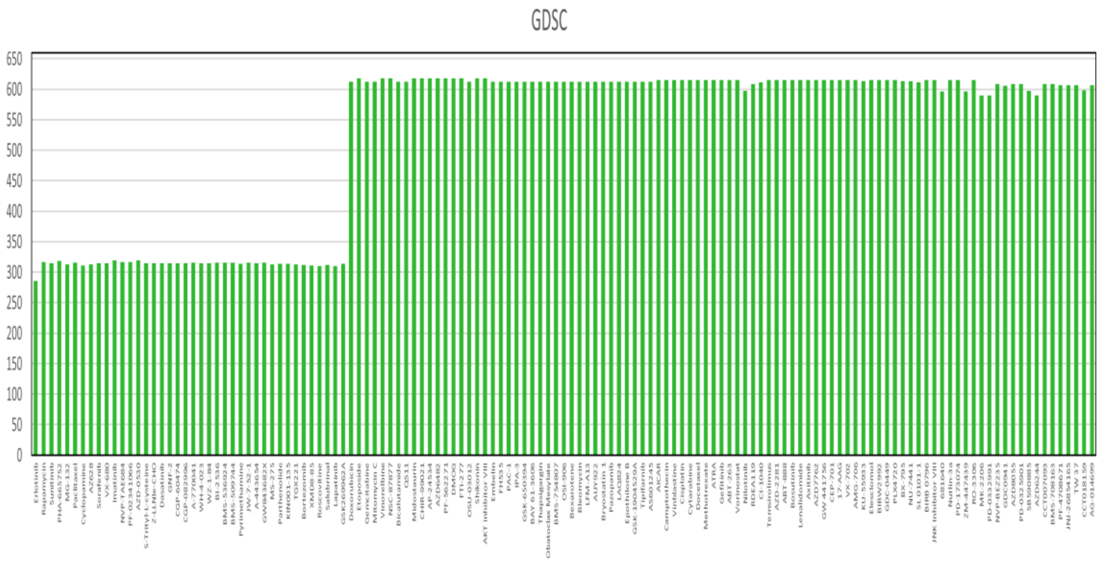


**Supplementary Figure 2.** The distribution of known responses for all drugs in CCLE.

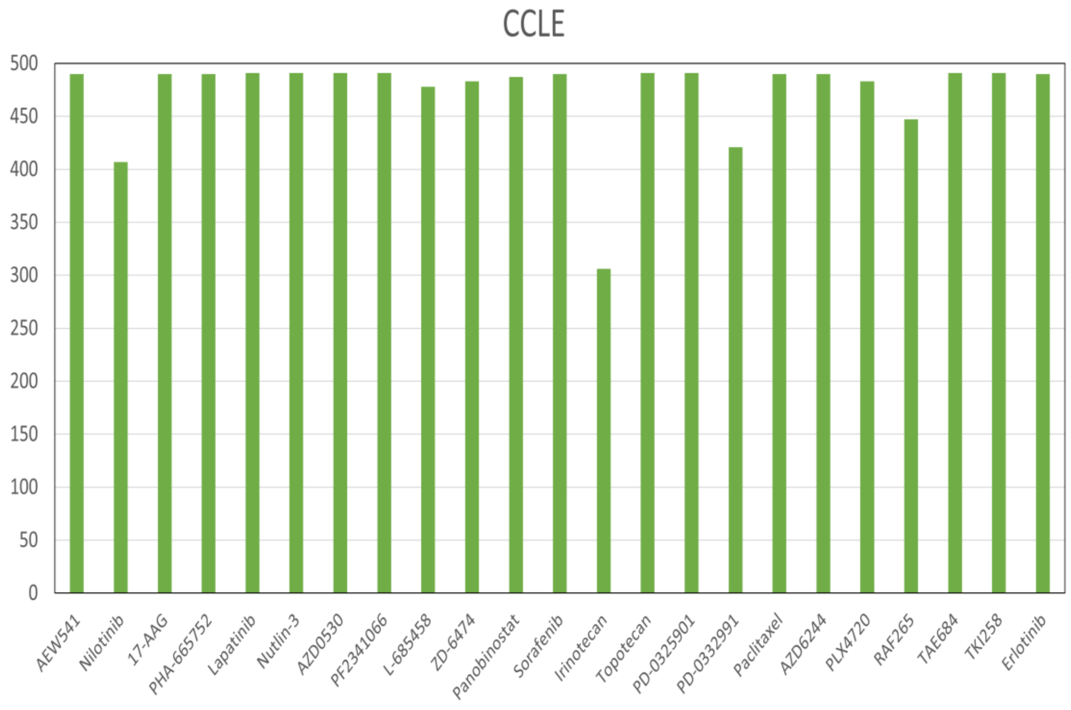


**Supplementary Table 1.** The ranked cell lines for each drug according to the predicted response values of WGRMF in GDSC. We applied WGRMF on GDSC dataset to predict the response values for those drug-cell line pairs without known

responses. The cell lines were ranked according to the predicted response values for each drug in GDSC, and the top 20 cell lines were selected for each drug. This prediction result is released for further experimental validation and research.
