# Supplementary material for: Anticancer Drug Response Prediction in Cell Lines Using Weighted Graph Regularized Matrix Factorization
Source: Mol Ther Nucleic Acids. 2019 Jun 4;17:164–74. doi: 10.1016/j.omtn.2019.05.017 (PMC6610642; doi:10.1016/j.omtn.2019.05.017)
Supplement: Document S2. Article plus Supplemental Information [file mmc2.pdf]

Molecular Therapy
Nucleic Acids
Original Article

# Anticancer Drug Response Prediction in Cell Lines Using Weighted Graph Regularized Matrix Factorization

Na-Na Guan,[1] Yan Zhao,[2] Chun-Chun Wang,[2] Jian-Qiang Li,[1] Xing Chen,[2] and Xue Piao[3]

[1]College of Computer Science and Software Engineering, Shenzhen University, Shenzhen 518060, China; [2]School of Information and Control Engineering, China University of Mining and Technology, Xuzhou 221116, China; [3]School of Medical Informatics, Xuzhou Medical University, Xuzhou 221004, China

**Precision medicine has become a novel and rising concept, which depends much on the identification of individual genomic signatures for different patients. The cancer cell lines could reflect the "omic" diversity of primary tumors, based on which many works have been carried out to study the cancer biology and drug discovery both in experimental and computational aspects. In this work, we presented a novel method to utilize weighted graph regularized matrix factorization (WGRMF) for inferring anticancer drug response in cell lines. We constructed a *p*-nearest neighbor graph to sparsify drug similarity matrix and cell line similarity matrix, respectively. Using the sparsified matrices in the graph regularization terms, we performed matrix factorization to generate the latent matrices for drug and cell line. The graph regularization terms including neighbor information could help to exclude the noisy ingredient and improve the prediction accuracy. The 10-fold cross-validation was implemented, and the Pearson correlation coefficient (PCC), root-mean-square error (RMSE), PCCsr, and RMSEsr averaged over all drugs were calculated to evaluate the performance of WGRMF. The results on the Genomics of Drug Sensitivity in Cancer (GDSC) dataset are 0.64 ± 0.16, 1.37 ± 0.35, 0.73 ± 0.14, and 1.71 ± 0.44 for PCC, RMSE, PCCsr, and RMSEsr in turn. And for the Cancer Cell Line Encyclopedia (CCLE) dataset, WGRMF got results of 0.72 ± 0.09, 0.56 ± 0.19, 0.79 ± 0.07, and 0.69 ± 0.19, respectively. The results showed the superiority of WGRMF compared with previous methods. Besides, based on the prediction results using the GDSC dataset, three types of case studies were carried out. The results from both cross-validation and case studies have shown the effectiveness of WGRMF on the prediction of drug response in cell lines.**

## INTRODUCTION

Benefiting from the development of high-throughput sequencing technology and the improvement of bioinformatics, the precision medicine has become a novel and burgeoning concept.[1] The goal of precision medicine is to effectively classify the different states and processes for the same disease, and personally make accurate treatment for the individual patient.[2] It is critical for the success of precision medicine to identify individual genomic signatures for different patients.[3] Cancer is one of the most threatening human complex diseases. The cancer cell lines could reflect the "omic" diversity of primary tumors, which therefore could be considered as a promising proxy to characterize the therapeutic response.[4] Using cell lines, many works have been carried out to study the cancer biology and drug discovery both in experimental and computational aspects.[5–7] As the basis of in-depth researches, tremendous genomic and pharmacological data have been collected and categorized in large scale for diverse cancer cell lines.[4,8–12] The consequential mission is to develop powerful methods to extract useful information from those complicated datasets and find the connections between the cancer information and the drug response.

Because experiments are expensive and time-consuming, computational approaches have been developed for tumorous drug response study, which are attracting more and more researchers' attentions.[13–15] There are mainly two classes of computational models to predict drug response in cancer cell lines. The first one is based on machine learning algorithms, such as elastic-net regression,[16] support vector machine (SVM), and random forest (RF).[17] For instance, Menden et al.[18] proposed a machine learning method for drug sensitivity inference, which utilized neural network and RF as predictors. In their model, they combined cell line genomic features and drug chemical structures to be the input features, and collected response data from the Genomics of Drug Sensitivity in Cancer (GDSC) project as the training samples. The prediction accuracy of the method in 8-fold cross-validation was improved because of the integration of information from both cell lines and drugs. Besides, Fersini et al.[19] presented a computational framework using the Consensus p-Median clustering approach for drug response inference in tumor cell lines. They

Received 25 February 2019; accepted 20 May 2019;
https://doi.org/10.1016/j.omtn.2019.05.017.

**Correspondence:** Xue Piao, School of Medical Informatics, Xuzhou Medical University, Xuzhou 221004, China.
**E-mail:** px@xzhmu.edu.cn
**Correspondence:** Xing Chen, School of Information and Control Engineering, China University of Mining and Technology, Xuzhou 221116, China.
**E-mail:** xingchen@amss.ac.cn
**Correspondence:** Jian-Qiang Li, College of Computer Science and Software Engineering, Shenzhen University, Shenzhen 518060, China.
**E-mail:** lijq@szu.edu.cn

 © 2019 The Author(s).
This is an open access article under the CC BY license (http://creativecommons.org/licenses/by/4.0/).

performed the Consensus p-Median clustering algorithm to create homogeneous groups of tumor cell lines, based on which the relevant genes that could be responsible for drug responses were selected to characterize the cell line clusters. Then, the Bayesian networks were constructed for the prediction of different drug responses for cell lines with given genomic profiles. Geeleher et al.[20] proposed a framework that adopted only the before-treatment gene expression profile to predict the drug response. After the gene expressions of cell line and clinical trial were combined and homogenized, the linear ridge regression was trained and tested, which could be implemented to predict drug sensitivity for new cell lines. Furthermore, Tan et al.[21] devised a classifier based on SVM with pairwise kernels for the classification of drug responses. The chemical compound was represented with labeled undirected graph and fingerprint, whereas the cell line was characterized using gene transcriptional profile, gene copy number, gene mutational status, and microRNA expression information. Based on diverse features of drug and cell line, several different kernels, especially the pairwise kernel, were employed through computing drug similarity and cell line similarity in different ways. Similarly, combining various similarity information, Wang et al.[22] presented a model called Predict Drug Response in Cancer Cells (PDRCC) to infer novel drug responses in cancer cells. The PDRCC calculated several drug similarities and cell line similarities based on diverse feature information. Then, they calculated the Kronecker product of any similarity of drug and cell line as the drug-cell line pair kernel function, which was sequentially fed to SVM to predict drug responses. Besides, based on SVM and a recursive feature selection tool, Dong et al.[23] developed a predictor, using gene expression of cancer cell and drug response data in the Cancer Cell Line Encyclopedia (CCLE). They classified the cell lines into sensitive and resistant subsets according to their responses to each drug. Then, a wrapper method named Support Vector Machine Recursive Feature Elimination (SVM-RFE) was used for the feature selection. The classified responses and the selected features from CCLE data were finally input to the SVM model for training and predicting.

As is known, the training dataset and the feature of samples are vitally important to machine learning models. To solve the problem of different source domains of the training and test datasets, Turki et al.[24] presented a transfer learner for inferring drug response in cancer cell lines, using mean shift and Procrustes analysis. Honkela et al.[25] proposed an approach that integrated differentially private learning and Bayesian linear regression, for drug sensitivity prediction with limited dataset. As a subtype of transfer learning, the multi-task learning models have shown their capability for drug response inference. For example, Gönen et al.[26] developed a Kernelized Bayesian Multi-task Learning (KBMTL) for inference of drug response in cell lines, which employed kernel-based dimension reduction. Besides, Tan[27] presented an approach using multi-task learning regularized with trace-norm to improve the kernelized transfer learning for anticancer drug response prediction. Similarly, Yuan et al.[28] also developed a multi-task learning method with trace-norm regularization, in which an efficient optimization algorithm called alternating direction method of multipliers (ADMM) was adopted.

Ensemble learning has been widely used in prediction problems of bioinformatics such as disease-specific risk variant prediction[29] and disease-related non-coding RNA prediction.[30–35] For drug response prediction, Tan et al.[36] proposed an Ensemble Learning for Drug Activity Prediction (ELDAP), which incorporated elastic net regression, KBMLT, pairwise support vector regressor (PSVR), and neural networks. Recently, Matlock et al.[37] studied the effect of stacking three different machine learning algorithms that included RF, deep learning (DL), and *k*-nearest neighbor (KNN) for inference of drug response in cancer cell lines. The results revealed the ability of the ensemble models to improve the prediction accuracy.

Within machine learning methods, matrix factorization is an efficient class of models for drug response inference. For example, Ammad-ud-din et al.[38] developed a method to extend quantitative structure-activity relationship (QSAR) analysis of drugs in cancer cell lines, which applied the kernelized Bayesian matrix factorization (KBMF) algorithm. They calculated kernel matrices for each type of feature for both drug and cell line through computing Jaccard coefficient or Gaussian kernel. Moreover, Wang et al.[39] recently proposed an improved method for drug response inference in cancer cell lines, which utilized the similarity regularized matrix factorization (SRMF).

Machine learning models could infer drug response in cell lines in large scale, which often neglected the topological information of drug similarity and cell line similarity. The network-based models could remedy the limitation. Shivakumar et al.[40] proposed a drug structural similarity-based model to predict drug sensitivity in cell lines, which assigned the sensitivity profile of the known drug to the new drug if they were structurally similar. Zhang et al.[41] established a dual-layer network composed of cell lines and drugs, utilizing the cell line similarity based on their gene expression profiles, and drug similarity based on their chemical structures. On the basis of the dual-layer network, they proposed a weighted method to predict the response of a cell line to a drug. Moreover, Kim et al.[42] predicted the drug response of cancer cell lines using a network-based classifier (NBC). In this method, a sensitive network and a resistant network were respectively constructed using selected genes, based on which linear and non-linear predictor functions were designed for gene expression prediction, which could be used to classify a new cell line via comparing the results from the sensitive and resistant networks. Turki et al.[43] utilized a link-filtering algorithm on the cell line network followed with regression algorithms to predict the cancer drug sensitivity. Moreover, Stanfield et al.[44] integrated the information of genes, cell lines, and drugs to construct a heterogeneous network, on which a link prediction with random walk with restart (RWR) was performed to build the network profiles of cell lines and drugs. Finally, the Pearson correlation coefficients (PCCs) between drugs and cell lines were calculated for drug-cell line association prediction. Recently, Zhang et al.[45] developed a model of Heterogeneous Network-based Method for Drug Response Prediction (HNMDRP) through integrating five subnetworks, including cell line similarity network, drug similarity network, target similarity network, drug-cell line association network, and

drug-protein network. An information flow-based algorithm was then implemented on the heterogenous network to predict novel drug-cell line associations. In addition, Le et al.[46] also constructed a heterogeneous network by combining omics-based cell line similarity, drug structural similarity, and known drug responses of cell lines, based on which a global method called GloNetDRP was implemented for drug response prediction. The RWR algorithm was adopted in this model to compute the response value of test cell lines to test drugs.

Previous computational methods often have their own limitations. For instance, some machine learning-based models predict only binary results; i.e., they classify cell line-drug pairs into sensitive or resistant groups. Some network-based models could be used to predict new drug-cell line associations, but cannot give the precision response values. And some other models have the need to improve their prediction accuracy. In this paper, we presented a novel approach to infer drug response in cancer cell lines using the weighted graph regularized matrix factorization (WGRMF) algorithm. For drug similarity and cell line similarity, a sparsification technique was operated using the $p$-nearest neighbor graphs that were constructed for drug and cell line, respectively, based on the KNN algorithm. The sparsified similarity matrices were then used to regularize the latent matrices learnt from matrix factorization together with Tikhonov regularization. Consequently, the latent drug vectors and latent cell line vectors could be obtained, respectively, through an alternative update operation. The predicted response matrix was finally computed via multiplying the two low-rank latent matrices. The performance of our model was evaluated in the 10-fold cross-validation through calculating the PCC and root-mean-square error (RMSE) between predicted values and observed values in two datasets, CCLE and GDSC. The results demonstrated the superiority of WGRMF compared with SRMF, which directly used the drug similarity and cell line similarity as regularization terms in the matrix factorization model.[39] In the case studies, we compared the predicted values of missing drug responses and the known response values in GDSC to investigate the correlation between drug sensitivity and several gene variations, such as lapatinib sensitivity and erlotinib sensitivity to the Epidermal Growth Factor Receptor (EGFR) gene, PD-0332991 sensitivity to Cyclin Dependent Kinase Inhibitor 2A (CDKN2A) gene, as well as pazopanib resistance of KRAS Proto-Oncogene, GTPase (KRAS). Moreover, we found that the results of WGRMF could help to identify new sensitive genes of drugs through combining predicted data with existing data. The association between MET Proto-Oncogene, Receptor Tyrosine Kinase (MET) gene, and PHA-665752 was an example for this. Besides, we applied WGRMF to the discovery of sensitive drugs for non-small cell lung cancer (NSCLC).

# RESULTS

## Performance Evaluation

We utilized 10-fold cross-validation to evaluate the performance of the proposed method in the CCLE dataset and GDSC dataset, respectively. Specifically, the known response values were randomly divided into 10 subsets with equal size. Then, one subset was left in turn as the test set and the other nine were combined as the training set. We can get the predicted response values of the test set through implementing WGRMF learnt from the training set. As soon as the loop ends, each of the 10 subsets was considered as the test sample one after another, and we could obtain the predicted value as the counterpart of each known response value. To improve the reliability of the results, the whole process of cross-validation was repeated a hundred times to estimate the average performance of the model. Based on the predicted and observed response data, we calculated the PCC and RMSE for each drug to estimate the capability of WGRMF on predicting drug response in cell lines. The PCC value indicates the extent of correlation between the predicted and observed response profiles of a drug, which could be formulated as

$$\mathrm{PCC} = \frac{\sum_{i=1}^{nd}(r_i - \overline{r})\left(\widehat{r}_i - \overline{\widehat{r}}\right)}{\sqrt{\sum_{i=1}^{nd}(r_i - \overline{r})^2 \sum_{i=1}^{nd}\left(\widehat{r}_i - \overline{\widehat{r}}\right)^2}}, \qquad \text{(Equation 1)}$$

where $r$ and $\widehat{r}$ indicate the original and predicted response values, respectively; $\overline{r}$ and $\overline{\widehat{r}}$ denote their mean values, respectively; and $nd$ is the total number of known response values for the query drug. The larger the PCC value is, the more accurate the prediction is. The RMSE stands for the deviation of predicted values from observed values, which is expected to be small. The RMSE for a drug could be calculated as

$$\mathrm{RMSE} = \sqrt{\frac{\sum_{i=1}^{nd}(r_i - \widehat{r}_i)^2}{nd}}. \qquad \text{(Equation 2)}$$

After the PCC and RMSE were calculated for all drugs, we correspondingly computed the average PCC and average RMSE over drugs.

In further estimation of the WGRMF performance, we focused on the sensitive and resistant cell lines for each drug. First, we ranked cell lines for each drug according to the response values and split them into four equal parts. Then the first and last parts were selected to compose the sensitive and resistant cell line set of each drug. Consequently, we could obtain the PCC and RMSE of sensitive and resistant cell lines for each drug, as well as the average values of PCC and RMSE.

For convenience, we used PCC and RMSE to indicate the results from all cell lines, and PCCsr and RMSEsr to indicate the results from sensitive and resistant cell lines. As a result, the WGRMF got PCC, RMSE, PCCsr, and RMSEsr averaged over 23 drugs in CCLE as 0.72 ± 0.09, 0.56 ± 0.19, 0.79 ± 0.07, and 0.69 ± 0.19, respectively. For data in GDSC, the results of WGRMF are 0.64 ± 0.16, 1.37 ± 0.35, 0.73 ± 0.14, and 1.71 ± 0.44 for PCC, RMSE, PCCsr, and RMSEsr averaged over 135 drugs. To make a comparison with SRMF, the same process of 10-fold cross-validation was carried out using SRMF on both CCLE and GDSC datasets. It is worth noting that all of the above results refer to the average values and the

corresponding SDs for a hundred experiments of cross-validation. The overall results of comparing WGRMF with SRMF can be seen from Table 1, which shows that the performance of WGRMF is superior to SRMF based on all indicators for both CCLE and GDSC datasets.

Furthermore, in order to inspect how the WGRMF performed on individual drugs, we selected drugs targeting genes in the phosphatidylinositol-4,5-bisphosphate 3-kinase (PI3K) pathway from the GDSC dataset as examples. PI3K is well known as a signaling component downstream of receptor tyrosine kinases (RTKs), which plays important roles in various biological responses.[47,48] The comparisons between WGRMF and SRMF on PCCsr and RMSEsr were illustrated in Figures 1 and 2, respectively. From the histograms, we can see that the results of WGRMF are better than that of SRMF for most of the PI3K pathway drugs.

### Case Studies

In this work, we have used all existing response values in the GDSC dataset to train the model, which was sequentially adopted to predict the response values for those drug-cell line pairs without known response values. Then, we ranked the cell lines for each drug in GDSC according to the predicted response values, and the top 20 most likely sensitive cell lines were selected for each drug. The ranked and selected result could be obtained from Table S1. This prediction result was released to provide some assistance for further experimental research. Furthermore, based on the whole predicted response of the GDSC dataset, we have conducted three types of case studies, including consistency identification for drug sensitivity of gene mutation, novel drug-gene association discovery, and drug reposition on specific cancer type.

In the first case of consistency identification, we made a comparative analysis between the predicted and the observed responses of four drugs, based on several gene mutation profiles of cell lines. Lapatinib, known as a small-molecule kinase inhibitor, could target the EGFR gene and Erb-B2 Receptor Tyrosine Kinase 2 (ERBB2) gene, which was approved by the US Food and Drug Administration (FDA) in 2007 for the therapy of breast cancer patients.[49] Dual inhibition of EGFR and ERBB2 tyrosine kinases plays a clinical biological role in suppressing the proliferation and survival of those cancer cells that are promoted by signaling pathways.[50–52] For lapatinib, the responses in 310 cell lines are known, whereas 342 cell lines are without known responses. The EGFR mutation profile of cell lines was used to investigate the association between the gene mutation and the response to lapatinib. The predicted responses to lapatinib were classified into the EGFR mutation group and EGFR wild-type group, and similar operation was exerted on the observed responses. All of the four groups of responses were plotted in one figure to identify the consistency between predicted and existing datasets for lapatinib sensitivity in EGFR mutation cell lines. The comparative result was shown in Figure 3A, from which we can see that EGFR-mutated cell lines are more sensitive to lapatinib for both predicted and observed data. Erlotinib, a kind of OSI Pharmaceuticals, has been reported as an inhibitor of the tyrosine kinase activity of EGFR,[53,54] which is efficiently used to prolong the lifetime of the previously treated patients with NSCLC.[55,56] In the GDSC dataset, there are 286 out of 652 cell lines with known response values to erlotinib. In this work, we also verified the sensitivity of EGFR mutation to erlotinib. The same trend could be observed between the predicted and existing data, which was shown in Figure 3B. Furthermore, PD-0332991, also named palbociclib, with 590 existing response values in cell lines in GDSC, is an effective drug in the treatment of several cancers including breast cancer, as a cyclin-dependent kinase 4/6 inhibitor.[57–59] The CDKN2A mutation was selected to investigate its contribution to the PD-0332991 sensitivity. By comparing the predicted result of WGRMF with the existing data, we can identify the consistency between them, which is given in Figure 3C. Besides, the resistance of gene mutation to drug also could be predicted by the WGRMF. Taking the response of KRAS mutated cell lines to pazopanib as an example, we can see the resistant tendency both from the known and the predicted results, which is shown in Figure 3D. In order to show the comparison results more distinctly, the rank-sum test was performed between the mutated and wild groups for both predicted and observed datasets. The calculated p values have been shown in each panel of Figure 3. From the results of the case study for drugs mentioned above, we could observe the agreement of the predicted responses to the known responses in the GDSC dataset, based on the drug sensitivity of gene mutation profiles.

In the second case study, we used the predicted responses of WGRMF integrated with the known data in GDSC to infer potential drug-cancer gene associations. For instance, PHA-665752 was

**Table 1. The Comparison Results between WGRMF and SRMF under the 10-Fold Cross-Validation in CCLE and GDSC Datasets, Indicated by Drug-Averaged PCC, RMSE, PCCsr, and RMSEsr**

| Dataset | Model | Drug-Averaged PCC | Drug-Averaged RMSE | Drug-Averaged PCCsr | Drug-Averaged RMSEsr |
|---|---|---|---|---|---|
| CCLE | WGRMF | 0.72 ± 0.09 | 0.56 ± 0.19 | 0.79 ± 0.07 | 0.69 ± 0.19 |
| | SRMF | 0.71 ± 0.09 | 0.57 ± 0.18 | 0.78 ± 0.08 | 0.74 ± 0.22 |
| GDSC | WGRMF | 0.64 ± 0.16 | 1.37 ± 0.35 | 0.73 ± 0.14 | 1.71 ± 0.44 |
| | SRMF | 0.61 ± 0.16 | 1.52 ± 0.36 | 0.71 ± 0.14 | 1.79 ± 0.45 |

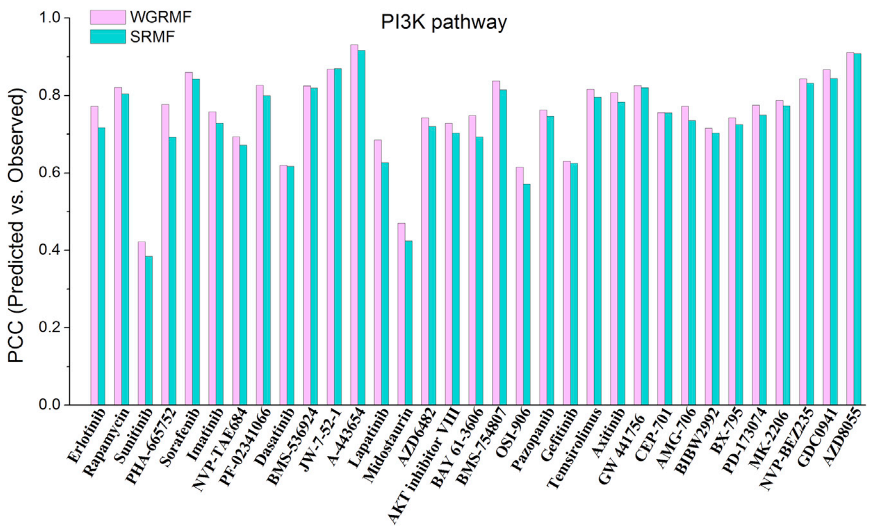


**Figure 1. The Comparison Histogram of PCCsr for the Drugs That Target Genes in PI3K Pathway**

The comparison histogram of PCCsr between WGRMF and SRMF under the 10-fold cross-validation for the drugs that target genes in the PI3K pathway.

such a small molecule that it could act as the ATP competitive inhibitor of the catalytic activity of c-Met kinase.[60,61] It has been reported that PHA-665752 could act on the growth and motility of multiple cancer cell lines.[62–64] In the GDSC dataset, the number of cell lines with known response to PHA-665752 is 381, out of which only two cell lines are related to MET amplification. It is difficult to observe the tendency of drug sensitivity based on the scarce available data. After combining the newly predicted responses and the existing data, we could obtain the sensitivity of MET amplification to PHA-665752 (see Figure 4). The p values obtained from the rank-sum test were also given in Figure 4. The extreme susceptibility of MET amplification to the PHA-665752 has been experimentally confirmed in gastric cancer cell lines.[65]

Furthermore, as the third case study, we used the predicted responses of WGRMF to reposition drugs on specific cancer through combining the existing responses. For a given drug, we compared the responses of cell lines with a specific tissue type with the responses of other cell lines, which would help to find some useful information of the drug sensitivity on the specific cancer type. Lung cancer is one of the most common malignant tumors in the world, of which about 80% is NSCLC.[66] Gefitinib is the common drug to treat NSCLC patients with mutated EGFR.[67] Based on the predicted results of WGRMF combined with known responses in GDSC, we screened drugs for drug reposition on NSCLC. For example, through analyzing the response difference between NSCLC cell lines and other cell lines to PHA-665752, we found that NSCLC cell lines were more sensitive to PHA-665752 based on the integrated result, which could not be observed by considering only the existing data. The comparison among predicted, existing, and combined results and the p values that were computed using the rank-sum test between the NSCLC group and the other group were illustrated in Figure 5. It has been reported that the PHA-665752 could act as a c-MET inhibitor to prevent K-ras mutant NSCLC[60,68] or to treat NSCLC by cooperating with rapamycin.[69]

## DISCUSSION

The work to investigate drug response in cancer cell lines is crucial for the precision therapy of cancer patients. In this work, we presented a novel method to utilize WGRMF for inferring anticancer drug response in cell lines, which combined the information of drug similarity, cell line similarity, and known drug responses in cell lines. A sparsification technique was employed on the similarity matrices to construct the $p$-nearest neighbor graphs for drug and cell line, respectively. Then based on the low-rank approximation (LRA), together with Tikhonov regularization, drug graph regularization, and cell line graph regularization, the objective function of WGRMF was constructed, which could be solved through an alternative update rule to obtain the latent feature matrices for drug and cell line. The predicted response matrix was finally composed by the two latent matrices. To evaluate the performance of WGRMF, we implemented 10-fold cross-validation on two different datasets, GDSC and CCLE. The averaged PCC, RMSE, PCCsr, and RMSEsr over all drugs in each dataset were calculated as the measurement of model performance. In addition, several case studies based on the GDSC dataset were carried out. Just like its good performance on other issues, the WGRMF method has shown its excellent activity in predicting novel drug responses in cancer cell lines, which is better than SRMF in comparison.

There are several factors that could contribute to the superior performance of WGRMF. First of all, WGRMF was constructed on the local invariance assumption; that is, two close points in the original space will be close in the learnt manifolds in latent space. The postulate was guaranteed by the graph regularization with $p$-nearest neighbor graphs of drug and cell line. Besides, WGRMF took full advantage of neighborhood information via performing graph regularization. Furthermore, the sparsification process could help to eliminate the redundant and noisy information in the similarity matrices. Finally, the introduction of weight matrix into the objective function made it possible to consider the contribution from known responses only rather than from those unknown ones.

There are still some limitations existing in the current model. For example, the sparsification process and the LRA-based graph regularization have many parameters to choose or confirm. It is still

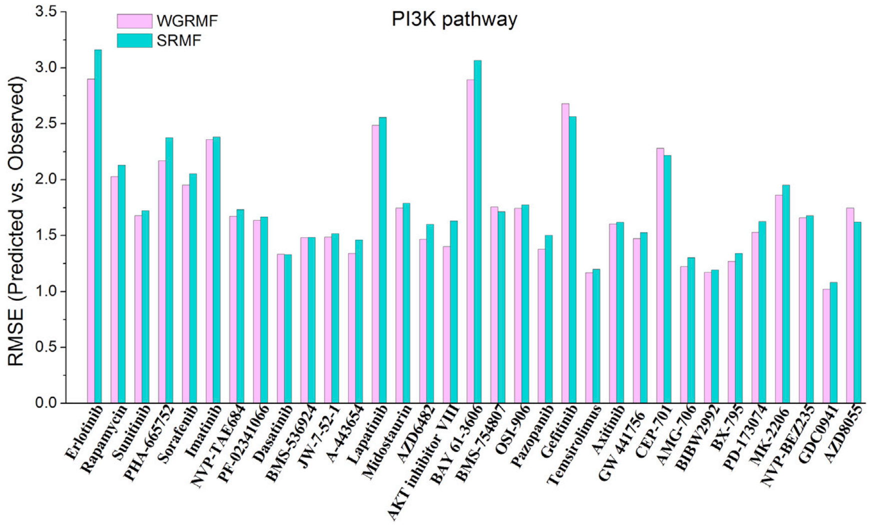


**Figure 2. The Comparison Histogram of RMSEsr for the Drugs That Target Genes in PI3K Pathway**

The comparison histogram of RMSEsr between WGRMF and SRMF under the 10-fold cross-validation for the drugs that target genes in the PI3K pathway.

difficult to optimize these parameters, which limits the application of this method. In addition, the cell line similarity was constructed only on the information of gene expression. If more genomic information of cell lines is incorporated in the cell line similarity, the prediction efficiency will be improved. In fact, some studies have demonstrated the effectiveness and robustness of the network framework based on diverse cancer hallmarks in cancer researches.[70,71] Thus, in future study, employing more cancer hallmarks to effectively construct the feature network of cell lines is imperative for drug response prediction. Recent researches indicated that both proteins and long non-coding RNAs (lncRNAs) could be used as a drug target.[72–76] Therefore, making full use of predicted and experimentally validated drug-target interactions involving both protein targets and non-coding RNA targets would benefit the prediction of drug response. Third, the lack of known responses for some drugs also restricts the performance of WGRMF. We expect that more response data can be collected from experimental results, which will improve the accuracy of the model. Besides, the optimal solution obtained from the alternative iteration is not the analytic solution, which may affect the prediction precision. In addition, predicting the response of drug combination would be an interesting and important direction for future studies.[77] Finally, the results in case studies were confirmed by only some published literature, which limited the scale of verification on the prediction results. In further research, more independent datasets derived from different databases should be applied to the validation of prediction results. This will make it more feasible and more authentic to evaluate the model performance.

## MATERIALS AND METHODS

### Genomic of Drug Sensitivity in Cancer Project

The first dataset we used was collected from Genomic of Drug Sensitivity in Cancer project (release-5.0, https://www.cancerrxgene.org/downloads), including 652 cancer cell lines, 135 drugs, and 70,676 known response values.[11,12] The distribution of known responses for all drugs has been shown in Figure S1, from which we can see that most of the drugs have more than 90% observed values, and only 40 drugs have known responses in less than 50% of cell lines. In GDSC, the drug sensitivity in the cancer cell line is measured by log-transformed $IC_{50}$ value that indicates the drug concentration for 50% inhibition *in vitro*. The more sensitive cell lines will get lower $IC_{50}$ values to a drug. Furthermore, for the involved drugs and cell lines, we need to characterize them with some features. Thus, we adopted the PubChem fingerprint descriptors as the features of the 135 drugs, which could be acquired from https://pubchem.ncbi.nlm.nih.gov. For those 652 cell lines, the gene expression profiles were taken to characterize them. Then, based on the features of drugs and cell lines, we can compute the drug similarity matrix and the cell line similarity matrix, respectively. Motivated by the method used in SRMF,[39] the Jaccard coefficient was utilized to calculate the drug similarity, and the PCC was computed to indicate the cell line similarity.

### CCLE

The second dataset was collected from the CCLE (https://portals.broadinstitute.org/ccle), which contains 23 drugs and 491 cell lines with 10,870 known responses.[4] Figure S2 gives the distribution of known responses over the 23 drugs. It is obvious that most of the drugs except 4 have more than 450 known responses. In CCLE, the response value of cell line to drug represents the area over the drug response curve, which is called the activity area. The larger values of activity area stand for more sensitive responses. Similar to the method used in GDSC, we then calculated Jaccard coefficients between drugs to measure the drug similarity based on the drug fingerprints. And for cell lines, the similarity was computed using PCC between gene expression profiles of cell lines.

### WGRMF

In this paper, inspired by the work of Ezzat et al.,[78] we have proposed a novel method using WGRMF for inferring anticancer drug response in cell lines (see Figure 6). We first represented the drug similarity, cell line similarity, as well as drug response in cell lines with matrix form, which were indicated by $S_d \in \mathbb{R}^{n \times n}$, $S_c \in \mathbb{R}^{m \times m}$, and $R \in \mathbb{R}^{n \times m}$, respectively. Before we implemented the WGRMF, we conducted a sparsification technique for the drug similarity matrix and cell line

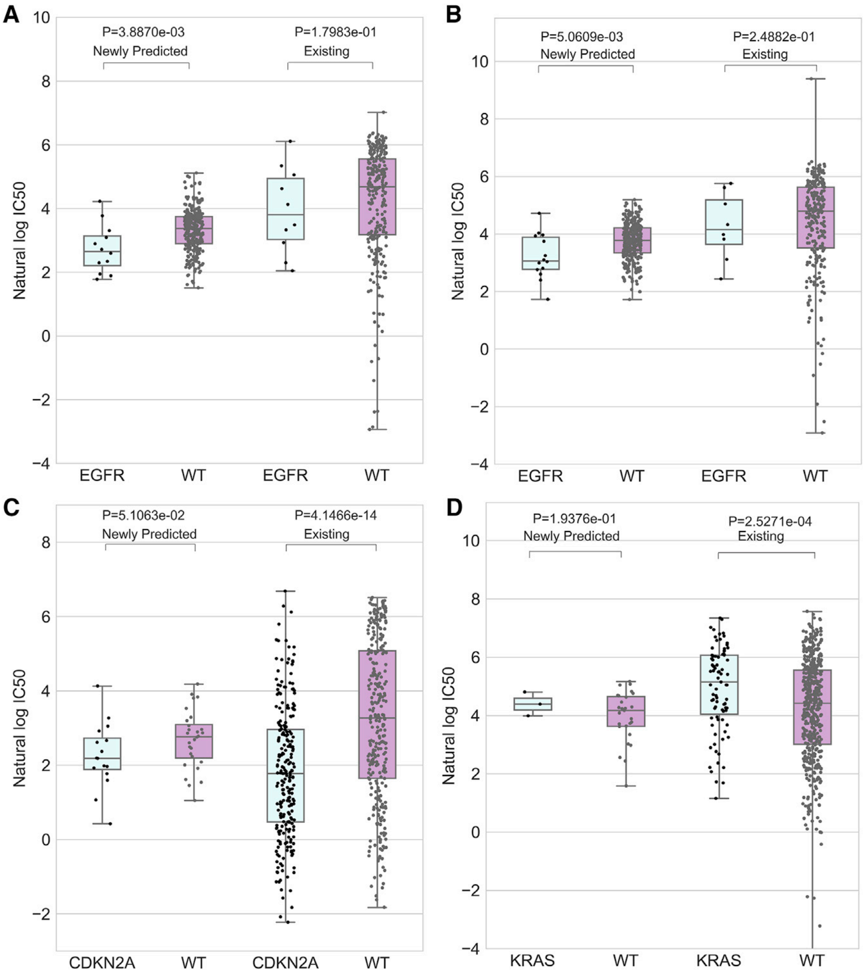


**Figure 3. The Results for Consistency Identification between Predicted and Existing Data for Four Drug-Gene Pairs Based on the GDSC Dataset**

(A) The responses of EGFR mutated and wild-type cell lines to lapatinib are shown. (B–D) EGFR mutation and erlotinib (B), CDKN2A mutation and PD-0332991 (C), and KRAS mutation and pazopanib (D). The p values obtained from the rank-sum test have been shown in each panel.

similarity matrix, respectively. For drugs, a matrix $N_d$ representing the $p$-nearest neighbor graph was constructed from drug similarity as follows:

$$N_d(i,j) = \begin{cases} 1, & \text{if } j \in \mathcal{N}_p(i)\ \&\ i \in \mathcal{N}_p(j) \\ 0, & \text{if } j \notin \mathcal{N}_p(i)\ \&\ i \notin \mathcal{N}_p(j) \\ 1/2, & \text{otherwise} \end{cases}, \qquad \text{(Equation 3)}$$

where $\mathcal{N}_p(i)$ represents the set of $p$ nearest neighbors of drug $d_i$. Similarly, a matrix $N_c$ corresponding to the $p$-nearest neighbor graph of cell line was constructed from the cell line similarity. Consequently, we could use the $p$-nearest neighbor graphs to sparsify the drug similarity, $S_d$, and cell line similarity, $S_c$, respectively, as follows:

$$\widehat{S}_d(i,j) = N_d(i,j) \cdot S_d(i,j) \qquad \text{(Equation 4)}$$

$$\widehat{S}_c(i,j) = N_c(i,j) \cdot S_c(i,j), \qquad \text{(Equation 5)}$$

where $\widehat{S}_d$ and $\widehat{S}_c$ stand for the sparsified similarity matrices of drug and cell line, respectively.

Based on the sparsified similarity matrices of drug and cell line, WGRMF was carried out for the prediction of drug response in cell lines. The fundamental of WGRMF is LRA, which can be formulated as follows:[78]

$$\min_{A,B} \|R - AB^T\|_F^2, \qquad \text{(Equation 6)}$$

where the drug response matrix $R$ was decomposed into two low-rank latent feature matrices $A \in \mathbb{R}^{n \times k}$ and $B \in \mathbb{R}^{m \times k}$, for drug and cell line, respectively. The variables $n$, $m$, and $k$ indicate the number of drugs, cell lines, and latent features in turn. The operator $\|\bullet\|_F$ means the Frobenius norm.

In order to eliminate the effect of overfitting and exclude the contribution from unknown responses in matrix $R$, we added the Tikhonov and graph regularization terms, and introduced the weight matrix $W$ into Equation 6 to obtain the objective function of WGRMF as follows:

$$\min_{A,B} \|W \odot (R - AB^T)\|_F^2 + \lambda_l\left(\|A\|_F^2 + \|B\|_F^2\right) + \lambda_d \sum_{i,r=1}^{n} \widehat{S}_d(i,r)\|a_i - a_r\|^2 + \lambda_c \sum_{j,q=1}^{n} \widehat{S}_c(j,q)\|b_j - b_q\|^2, \qquad \text{(Equation 7)}$$

where $W$ has the same shape with $R$, if there is a known response value in $R(i,j)$, $W(i,j) = 1$, otherwise $W(i,j) = 0$; $\lambda_l$, $\lambda_d$, and $\lambda_c$ are positive parameters; and $a_i$ and $b_j$ indicate the $i$th and $j$th rows of latent matrices $A$ and $B$, respectively. Each term in the objective function reflects a different goal of the problem. The first term is to make the predicted result close to known response data. The second one, called Tikhonov regularization, is to minimize the norms of latent matrices $A$ and $B$. The third one indicates the graph regularization of drugs, with the purpose to make two neighbor drugs nearest in the latent space. The last term has the similar meaning to the third one, which is the graph regularization of cell lines. According to

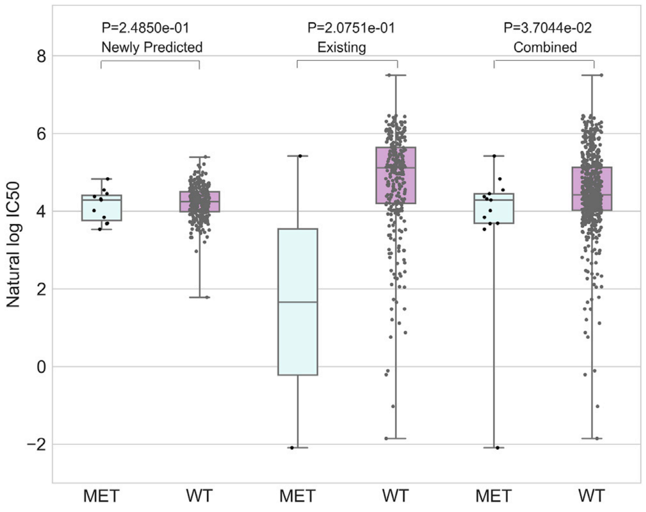


**Figure 4. The Responses of MET-Amplified and Wild-Type Cell Lines to PHA-665752 for Predicted, Existing, and Combined Data in GDSC**

The sensitivity of MET amplification to PHA-665752 could be obtained through combining the newly predicted responses and the existing data. The p values obtained from rank-sum test were given for predicted, existing, and combined data, respectively.

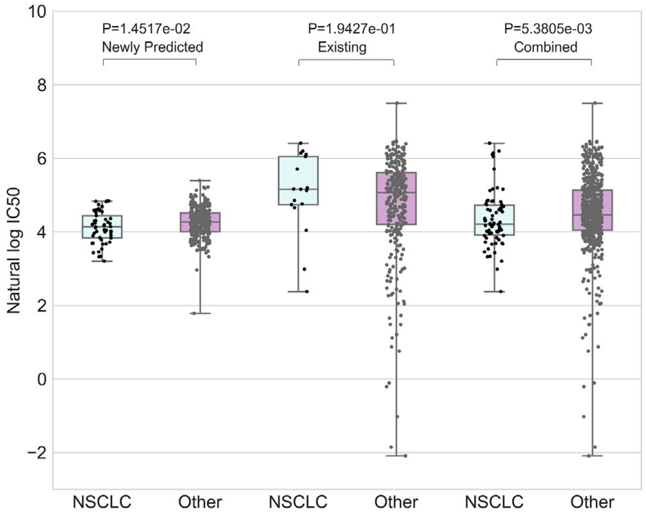


**Figure 5. The Reposition of PHA-665752 on NSCLC Based on the Combination of the Newly Predicted Responses and the Existing Data**

The figure shows that NSCLC cell lines are more sensitive to PHA-665752 based on the integrated result (p value of rank-sum test is $3.7044 \times 10^{-2}$), which could not be observed based only on existing data.

some previous studies,[78,79] the objective function in Equation 7 can be transformed into the following equation:

$$\min_{A,B} \left\| W \odot \left(R - AB^T\right) \right\|_F^2 + \lambda_l\left(\|A\|_F^2 + \|B\|_F^2\right) + \lambda_d Tr\left(A^T \mathcal{L}_d A\right) + \lambda_c Tr\left(B^T \mathcal{L}_c B\right), \quad \text{(Equation 8)}$$

where $Tr(\bullet)$ is the operator to calculate the trace of a matrix, and $\mathcal{L}_d$ and $\mathcal{L}_c$ indicate the graph Laplacians of sparsified similarity matrices $\widehat{S}_d$ and $\widehat{S}_c$, respectively, which are defined as follows:

$$\mathcal{L}_d = D_d - \widehat{S}_d \quad \text{(Equation 9)}$$

$$\mathcal{L}_c = D_c - \widehat{S}_c, \quad \text{(Equation 10)}$$

where $D_d$ and $D_c$ are two diagonal matrices derived from $\widehat{S}_d$ and $\widehat{S}_c$:

$$D_d(i,i) = \sum_r \widehat{S}_d(i,r) \quad \text{(Equation 11)}$$

$$D_c(j,j) = \sum_q \widehat{S}_c(j,q). \quad \text{(Equation 12)}$$

In order to improve the performance of the method, we utilized the normalized graph Laplacians to replace the unnormalized ones in Equation 8, which can be computed as follows:[78]

$$\tilde{\mathcal{L}}_d = D_d^{-1/2} \mathcal{L}_d D_d^{-1/2} \quad \text{(Equation 13)}$$

$$\tilde{\mathcal{L}}_c = D_c^{-1/2} \mathcal{L}_c D_c^{-1/2}. \quad \text{(Equation 14)}$$

Thereby the final objective function can be rewritten as follows:

$$\min_{A,B} \left\| W \odot \left(R - AB^T\right) \right\|_F^2 + \lambda_l\left(\|A\|_F^2 + \|B\|_F^2\right) + \lambda_d Tr\left(A^T \tilde{\mathcal{L}}_d A\right) + \lambda_c Tr\left(B^T \tilde{\mathcal{L}}_c B\right). \quad \text{(Equation 15)}$$

The optimal solutions of the above equation can be obtained by solving $(\partial L/\partial a_i) = 0$ and $(\partial L/\partial b_j) = 0$, where $L$ denotes the objective function in Equation 15, which finally results in two alternative update formulas:[78]

$$\forall i = 1 \ldots n, \quad a_i = \frac{\sum_{j=1}^m W(i,j) R(i,j) b_j - \lambda_d \tilde{\mathcal{L}}_d(i) A}{\sum_{j=1}^m W(i,j) b_j^T b_j + \lambda_l I_k} \quad \text{(Equation 16)}$$

$$\forall j = 1 \ldots m, \quad b_j = \frac{\sum_{i=1}^n W(i,j) R(i,j) a_i - \lambda_c \tilde{\mathcal{L}}_c(j) B}{\sum_{i=1}^n W(i,j) a_i^T a_i + \lambda_l I_k}. \quad \text{(Equation 17)}$$

We can get the latent matrices $A$ and $B$ row by row when the updates are converged. The predicted response matrix can be correspondingly computed as follows:

$$\widehat{R} = AB^T. \quad \text{(Equation 18)}$$

### Parameter Settings

We now show how to set the hyper-parameters used in the method in cross-validation and prediction. For the GDSC dataset,

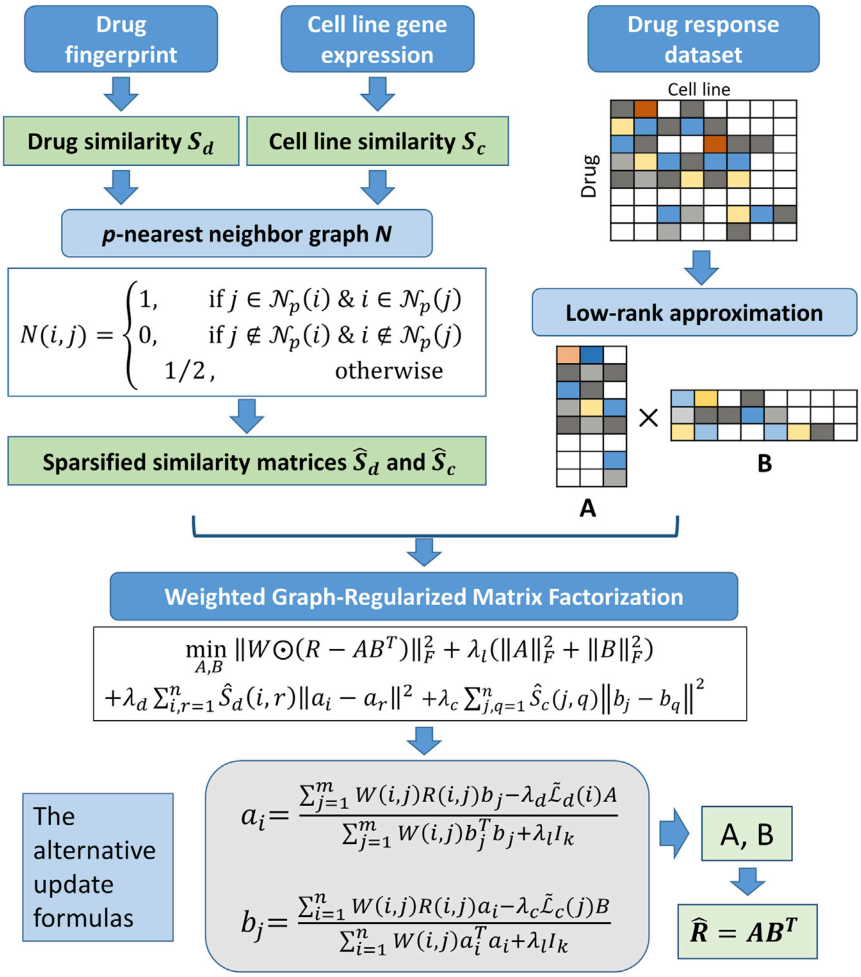


**Figure 6. The Flowchart of the WGRMF for Prediction of Drug Response in Cancer Cell Lines**
The flowchart of the WGRMF for prediction of drug response in cancer cell lines, based on the drug chemical structure similarity, cell line gene expression similarity, and known response data.

the sparsification parameter $p$ was set to $p = 20$ for both drug and cell line. The dimension parameter of latent matrix $k$ was chosen from {50, 100, min($n$, $m$)}. The three parameters, $\lambda_l$, $\lambda_d$, and $\lambda_c$, could be set using the grid search method, from the following values: $\lambda_l : \{2^{-2}, 2^{-1}, 2^0, 2^1\}$, $\lambda_d : \{2^{-5}, \ldots, 2^0, 2^1\}$, $\lambda_c : \{2^{-5}, \ldots, 2^0, 2^1\}$. As for the CCLE dataset, the value of $p$ in the sparsification process was set as $p = 10$ for both drug and cell line. The latent space dimension $k$ was set as $k = \min(n, m)$, because the value of min($n$, $m$) was <50.[78] $\lambda_l$, $\lambda_d$, and $\lambda_c$ were set in the same way as in the GDSC dataset.

## SUPPLEMENTAL INFORMATION

Supplemental Information can be found online at https://doi.org/10.1016/j.omtn.2019.05.017.

## AUTHOR CONTRIBUTIONS

N.-N.G. implemented the experiments, analyzed the result, and wrote the paper. X.C. conceived the project, developed the prediction method, designed the experiments, analyzed the result, revised the paper, and supervised the project. X.P., J.-Q.L., Y.Z., and C.-C.W. analyzed the result and revised the paper.

## CONFLICTS OF INTEREST

The authors declare no competing interests.

## ACKNOWLEDGMENTS

X.C. was supported by the National Natural Science Foundation of China under grant 61772531. J.-Q.L. was supported in part by the National Natural Science Foundation of China under grants U1713212, 61572330, and 61602319, and in part by the Research and Development Foundation from Shenzhen city, China, under grant CYZZ20160304165036893.

## REFERENCES

1. Collins, F.S., and Varmus, H. (2015). A new initiative on precision medicine. N. Engl. J. Med. *372*, 793–795.
2. Mirnezami, R., Nicholson, J., and Darzi, A. (2012). Preparing for precision medicine. N. Engl. J. Med. *366*, 489–491.
3. Ashley, E.A. (2015). The precision medicine initiative: a new national effort. JAMA *313*, 2119–2120.
4. Barretina, J., Caponigro, G., Stransky, N., Venkatesan, K., Margolin, A.A., Kim, S., Wilson, C.J., Lehár, J., Kryukov, G.V., Sonkin, D., et al. (2012). The Cancer Cell

Line Encyclopedia enables predictive modelling of anticancer drug sensitivity. Nature *483*, 603–607.

5. Heiser, L.M., Sadanandam, A., Kuo, W.L., Benz, S.C., Goldstein, T.C., Ng, S., Gibb, W.J., Wang, N.J., Ziyad, S., Tong, F., et al. (2012). Subtype and pathway specific responses to anticancer compounds in breast cancer. Proc. Natl. Acad. Sci. USA *109*, 2724–2729.

6. Cole, S.P., Bhardwaj, G., Gerlach, J.H., Mackie, J.E., Grant, C.E., Almquist, K.C., Stewart, A.J., Kurz, E.U., Duncan, A.M., and Deeley, R.G. (1992). Overexpression of a transporter gene in a multidrug-resistant human lung cancer cell line. Science *258*, 1650–1654.

7. Nagaraj, N., Wisniewski, J.R., Geiger, T., Cox, J., Kircher, M., Kelso, J., Pääbo, S., and Mann, M. (2011). Deep proteome and transcriptome mapping of a human cancer cell line. Mol. Syst. Biol. *7*, 548.

8. Hudson, T.J., Anderson, W., Artez, A., Barker, A.D., Bell, C., Bernabé, R.R., Bhan, M.K., Calvo, F., Eerola, I., Gerhard, D.S., et al.; International Cancer Genome Consortium (2010). International network of cancer genome projects. Nature *464*, 993–998.

9. Seashore-Ludlow, B., Rees, M.G., Cheah, J.H., Cokol, M., Price, E.V., Coletti, M.E., Jones, V., Bodycombe, N.E., Soule, C.K., Gould, J., et al. (2015). Harnessing Connectivity in a Large-Scale Small-Molecule Sensitivity Dataset. Cancer Discov. *5*, 1210–1223.

10. Iorio, F., Knijnenburg, T.A., Vis, D.J., Bignell, G.R., Menden, M.P., Schubert, M., Aben, N., Gonçalves, E., Barthorpe, S., Lightfoot, H., et al. (2016). A Landscape of Pharmacogenomic Interactions in Cancer. Cell *166*, 740–754.

11. Garnett, M.J., Edelman, E.J., Heidorn, S.J., Greenman, C.D., Dastur, A., Lau, K.W., Greninger, P., Thompson, I.R., Luo, X., Soares, J., et al. (2012). Systematic identification of genomic markers of drug sensitivity in cancer cells. Nature *483*, 570–575.

12. Yang, W., Soares, J., Greninger, P., Edelman, E.J., Lightfoot, H., Forbes, S., Bindal, N., Beare, D., Smith, J.A., Thompson, I.R., et al. (2013). Genomics of Drug Sensitivity in Cancer (GDSC): a resource for therapeutic biomarker discovery in cancer cells. Nucleic Acids Res. *41*, D955–D961.

13. Riddick, G., Song, H., Ahn, S., Walling, J., Borges-Rivera, D., Zhang, W., and Fine, H.A. (2011). Predicting in vitro drug sensitivity using Random Forests. Bioinformatics *27*, 220–224.

14. Frieboes, H.B., Edgerton, M.E., Fruehauf, J.P., Rose, F.R.A.J., Worrall, L.K., Gatenby, R.A., Ferrari, M., and Cristini, V. (2009). Prediction of drug response in breast cancer using integrative experimental/computational modeling. Cancer Res. *69*, 4484–4492.

15. Costello, J.C., Heiser, L.M., Georgii, E., Gönen, M., Menden, M.P., Wang, N.J., Bansal, M., Ammad-ud-din, M., Hintsanen, P., Khan, S.A., et al.; NCI DREAM Community (2014). A community effort to assess and improve drug sensitivity prediction algorithms. Nat. Biotechnol. *32*, 1202–1212.

16. Basu, A., Bodycombe, N.E., Cheah, J.H., Price, E.V., Liu, K., Schaefer, G.I., Ebright, R.Y., Stewart, M.L., Ito, D., Wang, S., et al. (2013). An interactive resource to identify cancer genetic and lineage dependencies targeted by small molecules. Cell *154*, 1151–1161.

17. Stetson, L.C., Pearl, T., Chen, Y., and Barnholtz-Sloan, J.S. (2015). Erratum: Computational identification of multi-omic correlates of anticancer therapeutic response. BMC Genomics *16*, 481.

18. Menden, M.P., Iorio, F., Garnett, M., McDermott, U., Benes, C.H., Ballester, P.J., and Saez-Rodriguez, J. (2013). Machine learning prediction of cancer cell sensitivity to drugs based on genomic and chemical properties. PLoS ONE *8*, e61318.

19. Fersini, E., Messina, E., and Archetti, F. (2014). A p-Median approach for predicting drug response in tumour cells. BMC Bioinformatics *15*, 353.

20. Geeleher, P., Cox, N.J., and Huang, R.S. (2014). Clinical drug response can be predicted using baseline gene expression levels and in vitro drug sensitivity in cell lines. Genome Biol. *15*, R47.

21. Tan, M. (2014). Drug sensitivity prediction for cancer cell lines based on pairwise kernels and miRNA profiles. In Proceeding of the IEEE International Conference on Bioinformatics and Biomedicine (BIBM), H. Zheng, X. Hu, D. Berrar, Y. Wang, W. Dubitzky, J.-K. Hao, K.-H. Cho, and D. Gilbert, eds. (IEEE), pp. 156–161.

22. Wang, Y., Fang, J., and Chen, S. (2016). Inferences of drug responses in cancer cells from cancer genomic features and compound chemical and therapeutic properties. Sci. Rep. *6*, 32679.

23. Dong, Z., Zhang, N., Li, C., Wang, H., Fang, Y., Wang, J., and Zheng, X. (2015). Anticancer drug sensitivity prediction in cell lines from baseline gene expression through recursive feature selection. BMC Cancer *15*, 489.

24. Turki, T., Wei, Z., and Wang, J.T.L. (2018). A transfer learning approach via procrustes analysis and mean shift for cancer drug sensitivity prediction. J. Bioinform. Comput. Biol. *16*, 1840014.

25. Honkela, A., Das, M., Nieminen, A., Dikmen, O., and Kaski, S. (2018). Efficient differentially private learning improves drug sensitivity prediction. Biol. Direct *13*, 1.

26. Gönen, M., and Margolin, A.A. (2014). Drug susceptibility prediction against a panel of drugs using kernelized Bayesian multitask learning. Bioinformatics *30*, i556–i563.

27. Tan, M. (2016). Prediction of anti-cancer drug response by kernelized multi-task learning. Artif. Intell. Med. *73*, 70–77.

28. Yuan, H., Paskov, I., Paskov, H., González, A.J., and Leslie, C.S. (2016). Multitask learning improves prediction of cancer drug sensitivity. Sci. Rep. *6*, 31619.

29. Chen, L., Jin, P., and Qin, Z.S. (2016). DIVAN: accurate identification of non-coding disease-specific risk variants using multi-omics profiles. Genome Biol. *17*, 252.

30. Chen, X., Zhou, Z., and Zhao, Y. (2018). ELLPMDA: Ensemble learning and link prediction for miRNA-disease association prediction. RNA Biol. *15*, 807–818.

31. Chen, X., Xie, D., Zhao, Q., and You, Z.H. (2019). MicroRNAs and complex diseases: from experimental results to computational models. Brief. Bioinform. *20*, 515–539.

32. Chen, X., Yan, C.C., Zhang, X., and You, Z.H. (2017). Long non-coding RNAs and complex diseases: from experimental results to computational models. Brief. Bioinform. *18*, 558–576.

33. Chen, X., and Huang, L. (2017). LRSSLMDA: Laplacian Regularized Sparse Subspace Learning for MiRNA-Disease Association prediction. PLoS Comput. Biol. *13*, e1005912.

34. Chen, X., Yin, J., Qu, J., and Huang, L. (2018). MDHGI: Matrix Decomposition and Heterogeneous Graph Inference for miRNA-disease association prediction. PLoS Comput. Biol. *14*, e1006418.

35. Chen, X., Wang, L., Qu, J., Guan, N.N., and Li, J.Q. (2018). Predicting miRNA-disease association based on inductive matrix completion. Bioinformatics *34*, 4256–4265.

36. Tan, M., Özgül, O.F., Bardak, B., Ekşioğlu, I., and Sabuncuoğlu, S. (2018). Drug response prediction by ensemble learning and drug-induced gene expression signatures. arXiv, arXiv:180203800v3, https://arxiv.org/abs/1802.03800.

37. Matlock, K., De Niz, C., Rahman, R., Ghosh, S., and Pal, R. (2018). Investigation of model stacking for drug sensitivity prediction. BMC Bioinformatics *19* (*Suppl 3*), 71.

38. Ammad-ud-din, M., Georgii, E., Gönen, M., Laitinen, T., Kallioniemi, O., Wennerberg, K., Poso, A., and Kaski, S. (2014). Integrative and personalized QSAR analysis in cancer by kernelized Bayesian matrix factorization. J. Chem. Inf. Model. *54*, 2347–2359.

39. Wang, L., Li, X., Zhang, L., and Gao, Q. (2017). Improved anticancer drug response prediction in cell lines using matrix factorization with similarity regularization. BMC Cancer *17*, 513.

40. Shivakumar, P., and Krauthammer, M. (2009). Structural similarity assessment for drug sensitivity prediction in cancer. BMC Bioinformatics *10* (*Suppl 9*), S17.

41. Zhang, N., Wang, H., Fang, Y., Wang, J., Zheng, X., and Liu, X.S. (2015). Predicting Anticancer Drug Responses Using a Dual-Layer Integrated Cell Line-Drug Network Model. PLoS Comput. Biol. *11*, e1004498.

42. Kim, S., Sundaresan, V., Zhou, L., and Kahveci, T. (2016). Integrating Domain Specific Knowledge and Network Analysis to Predict Drug Sensitivity of Cancer Cell Lines. PLoS ONE *11*, e0162173.

43. Turki, T., and Wei, Z. (2017). A link prediction approach to cancer drug sensitivity prediction. BMC Syst. Biol. *11* (*Suppl 5*), 94.

44. Stanfield, Z., Coşkun, M., and Koyutürk, M. (2017). Drug Response Prediction as a Link Prediction Problem. Sci. Rep. *7*, 40321.

45. Zhang, F., Wang, M., Xi, J., Yang, J., and Li, A. (2018). A novel heterogeneous network-based method for drug response prediction in cancer cell lines. Sci. Rep. *8*, 3355.

46. Le, D.H., and Pham, V.H. (2018). Drug Response Prediction by Globally Capturing Drug and Cell Line Information in a Heterogeneous Network. J. Mol. Biol. *430* (18 Pt A), 2993–3004.

47. Cantley, L.C. (2002). The phosphoinositide 3-kinase pathway. Science *296*, 1655–1657.

48. Franke, T.F., Kaplan, D.R., and Cantley, L.C. (1997). PI3K: downstream AKTion blocks apoptosis. Cell *88*, 435–437.

49. Moy, B., Kirkpatrick, P., Kar, S., and Goss, P. (2007). Lapatinib. Nat. Rev. Drug Discov. *6*, 431–432.

50. Burris, H.A., 3rd (2004). Dual kinase inhibition in the treatment of breast cancer: initial experience with the EGFR/ErbB-2 inhibitor lapatinib. Oncologist *9* (*Suppl 3*), 10–15.

51. Kim, J.W., Kim, H.P., Im, S.A., Kang, S., Hur, H.S., Yoon, Y.K., Oh, D.Y., Kim, J.H., Lee, D.S., Kim, T.Y., and Bang, Y.J. (2008). The growth inhibitory effect of lapatinib, a dual inhibitor of EGFR and HER2 tyrosine kinase, in gastric cancer cell lines. Cancer Lett. *272*, 296–306.

52. Wainberg, Z.A., Anghel, A., Desai, A.J., Ayala, R., Luo, T., Safran, B., Fejzo, M.S., Hecht, J.R., Slamon, D.J., and Finn, R.S. (2010). Lapatinib, a dual EGFR and HER2 kinase inhibitor, selectively inhibits HER2-amplified human gastric cancer cells and is synergistic with trastuzumab in vitro and in vivo. Clin. Cancer Res. *16*, 1509–1519.

53. Sridhar, S.S., Seymour, L., and Shepherd, F.A. (2003). Inhibitors of epidermal-growth-factor receptors: a review of clinical research with a focus on non-small-cell lung cancer. Lancet Oncol. *4*, 397–406.

54. Fukuoka, M., Yano, S., Giaccone, G., Tamura, T., Nakagawa, K., Douillard, J.Y., Nishiwaki, Y., Vansteenkiste, J., Kudoh, S., Rischin, D., et al. (2003). Multi-institutional randomized phase II trial of gefitinib for previously treated patients with advanced non-small-cell lung cancer (The IDEAL 1 Trial). J. Clin. Oncol. *21*, 2237–2246.

55. Shepherd, F.A., Rodrigues Pereira, J., Ciuleanu, T., Tan, E.H., Hirsh, V., Thongprasert, S., Campos, D., Maoleekoonpiroj, S., Smylie, M., Martins, R., et al.; National Cancer Institute of Canada Clinical Trials Group (2005). Erlotinib in previously treated non-small-cell lung cancer. N. Engl. J. Med. *353*, 123–132.

56. Pérez-Soler, R., Chachoua, A., Hammond, L.A., Rowinsky, E.K., Huberman, M., Karp, D., Rigas, J., Clark, G.M., Santabárbara, P., and Bonomi, P. (2004). Determinants of tumor response and survival with erlotinib in patients with non–small-cell lung cancer. J. Clin. Oncol. *22*, 3238–3247.

57. Morgan, D.O. (1995). Principles of CDK regulation. Nature *374*, 131–134.

58. Fry, D.W., Harvey, P.J., Keller, P.R., Elliott, W.L., Meade, M., Trachet, E., Albassam, M., Zheng, X., Leopold, W.R., Pryer, N.K., and Toogood, P.L. (2004). Specific inhibition of cyclin-dependent kinase 4/6 by PD 0332991 and associated antitumor activity in human tumor xenografts. Mol. Cancer Ther. *3*, 1427–1438.

59. Finn, R.S., Crown, J.P., Lang, I., Boer, K., Bondarenko, I.M., Kulyk, S.O., Ettl, J., Patel, R., Pinter, T., Schmidt, M., et al. (2015). The cyclin-dependent kinase 4/6 inhibitor palbociclib in combination with letrozole versus letrozole alone as first-line treatment of oestrogen receptor-positive, HER2-negative, advanced breast cancer (PALOMA-1/TRIO-18): a randomised phase 2 study. Lancet Oncol. *16*, 25–35.

60. Puri, N., Khramtsov, A., Ahmed, S., Nallasura, V., Hetzel, J.T., Jagadeeswaran, R., Karczmar, G., and Salgia, R. (2007). A selective small molecule inhibitor of c-Met, PHA665752, inhibits tumorigenicity and angiogenesis in mouse lung cancer xenografts. Cancer Res. *67*, 3529–3534.

61. Ma, P.C., Schaefer, E., Christensen, J.G., and Salgia, R. (2005). A selective small molecule c-MET Inhibitor, PHA665752, cooperates with rapamycin. Clin. Cancer Res. *11*, 2312–2319.

62. Crosswell, H.E., Dasgupta, A., Alvarado, C.S., Watt, T., Christensen, J.G., De, P., Durden, D.L., and Findley, H.W. (2009). PHA665752, a small-molecule inhibitor of c-Met, inhibits hepatocyte growth factor-stimulated migration and proliferation of c-Met-positive neuroblastoma cells. BMC Cancer *9*, 411.

63. Mukohara, T., Civiello, G., Davis, I.J., Taffaro, M.L., Christensen, J., Fisher, D.E., Johnson, B.E., and Jänne, P.A. (2005). Inhibition of the met receptor in mesothelioma. Clin. Cancer Res. *11*, 8122–8130.

64. Chattopadhyay, C., El-Naggar, A.K., Williams, M.D., and Clayman, G.L. (2008). Small molecule c-MET inhibitor PHA665752: effect on cell growth and motility in papillary thyroid carcinoma. Head Neck *30*, 991–1000.

65. Smolen, G.A., Sordella, R., Muir, B., Mohapatra, G., Barmettler, A., Archibald, H., Kim, W.J., Okimoto, R.A., Bell, D.W., Sgroi, D.C., et al. (2006). Amplification of MET may identify a subset of cancers with extreme sensitivity to the selective tyrosine kinase inhibitor PHA-665752. Proc. Natl. Acad. Sci. USA *103*, 2316–2321.

66. Herbst, R.S., Heymach, J.V., and Lippman, S.M. (2008). Lung cancer. N. Engl. J. Med. *359*, 1367–1380.

67. Lynch, T.J., Bell, D.W., Sordella, R., Gurubhagavatula, S., Okimoto, R.A., Brannigan, B.W., Harris, P.L., Haserlat, S.M., Supko, J.G., Haluska, F.G., et al. (2004). Activating mutations in the epidermal growth factor receptor underlying responsiveness of non-small-cell lung cancer to gefitinib. N. Engl. J. Med. *350*, 2129–2139.

68. Yang, Y., Wislez, M., Fujimoto, N., Prudkin, L., Izzo, J.G., Uno, F., Ji, L., Hanna, A.E., Langley, R.R., Liu, D., et al. (2008). A selective small molecule inhibitor of c-Met, PHA-665752, reverses lung premalignancy induced by mutant K-ras. Mol. Cancer Ther. *7*, 952–960.

69. Ma, P.C., Schaefer, E., Christensen, J.G., and Salgia, R. (2005). A selective small molecule c-MET Inhibitor, PHA665752, cooperates with rapamycin. Clin. Cancer Res. *11*, 2312–2319.

70. McGee, S.R., Tibiche, C., Trifiro, M., and Wang, E. (2017). Network Analysis Reveals A Signaling Regulatory Loop in the PIK3CA-mutated Breast Cancer Predicting Survival Outcome. Genomics Proteomics Bioinformatics *15*, 121–129.

71. Zaman, N., Li, L., Jaramillo, M.L., Sun, Z., Tibiche, C., Banville, M., Collins, C., Trifiro, M., Paliouras, M., Nantel, A., et al. (2013). Signaling network assessment of mutations and copy number variations predict breast cancer subtype-specific drug targets. Cell Rep. *5*, 216–223.

72. Chen, X., Yan, C.C., Zhang, X., Zhang, X., Dai, F., Yin, J., and Zhang, Y. (2016). Drug-target interaction prediction: databases, web servers and computational models. Brief. Bioinform. *17*, 696–712.

73. Chen, X., Guan, N.-N., Sun, Y.-Z., Li, J.-Q., and Qu, J. (2018). MicroRNA-small molecule association identification: from experimental results to computational models. Brief. Bioinform. *2018*, bby098.

74. Qu, J., Chen, X., Sun, Y.-Z., Li, J.-Q., and Ming, Z. (2018). Inferring potential small molecule-miRNA association based on triple layer heterogeneous network. J. Cheminform. *10*, 30.

75. Wang, C.C., Chen, X., Qu, J., Sun, Y.Z., and Li, J.Q. (2019). RFSMMA: A New Computational Model to Identify and Prioritize Potential Small Molecule-MiRNA Associations. J. Chem. Inf. Model. *59*, 1668–1679.

76. Qu, J., Chen, X., Sun, Y.Z., Zhao, Y., Cai, S.B., Ming, Z., You, Z.H., and Li, J.Q. (2019). In Silico Prediction of Small Molecule-miRNA Associations Based on the HeteSim Algorithm. Mol. Ther. Nucleic Acids *14*, 274–286.

77. Chen, X., Ren, B., Chen, M., Wang, Q., Zhang, L., and Yan, G. (2016). NLLSS: Predicting Synergistic Drug Combinations Based on Semi-supervised Learning. PLoS Comput. Biol. *12*, e1004975.

78. Ezzat, A., Zhao, P., Wu, M., Li, X.L., and Kwoh, C.K. (2017). Drug-target interaction prediction with graph regularized matrix factorization. IEEE/ACM Trans. Computat. Biol. Bioinform. *14*, 646–656.

79. Gu, Q., Zhou, J., and Ding, C.H.Q. (2010). Collaborative Filtering: Weighted Nonnegative Matrix Factorization Incorporating User and Item Graphs. Proceedings of the 10th Siam International Conference on Data Mining, SDM 2010 (SIAM), pp 199–210.

OMTN, Volume 17

## Supplemental Information

# Anticancer Drug Response Prediction in Cell Lines Using Weighted Graph Regularized Matrix Factorization

**Na-Na Guan, Yan Zhao, Chun-Chun Wang, Jian-Qiang Li, Xing Chen, and Xue Piao**

# Anticancer drug response prediction in cell lines using weighted graph regularized matrix factorization

Na-Na Guan[1], Yan Zhao[2], Chun-Chun Wang[2], Jian-Qiang Li[1,*], Xing Chen[2,*], Xue Piao[3,*]

[1]College of Computer Science and Software Engineering, Shenzhen University, Shenzhen, 518060, China

[2]School of Information and Control Engineering, China University of Mining and Technology, Xuzhou, 221116, China

[3]School of Medical Informatics, Xuzhou Medical University, Xuzhou, 221004, China

*Corresponding author

**Email**: px@xzhmu.edu.cn; xingchen@amss.ac.cn; lijq@szu.edu.cn

**Keywords**: drug response; cell line; graph regularization; matrix factorization; response prediction

## Supplementary Information

**Supplementary Figure 1.** The distribution of known responses for all drugs in GDSC.

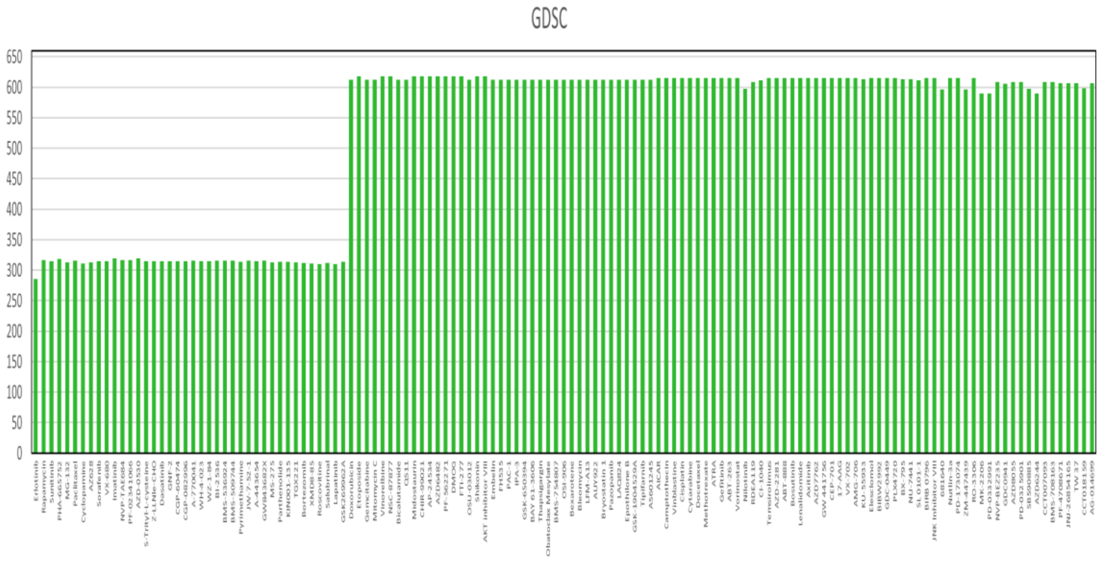


**Supplementary Figure 2.** The distribution of known responses for all drugs in CCLE.

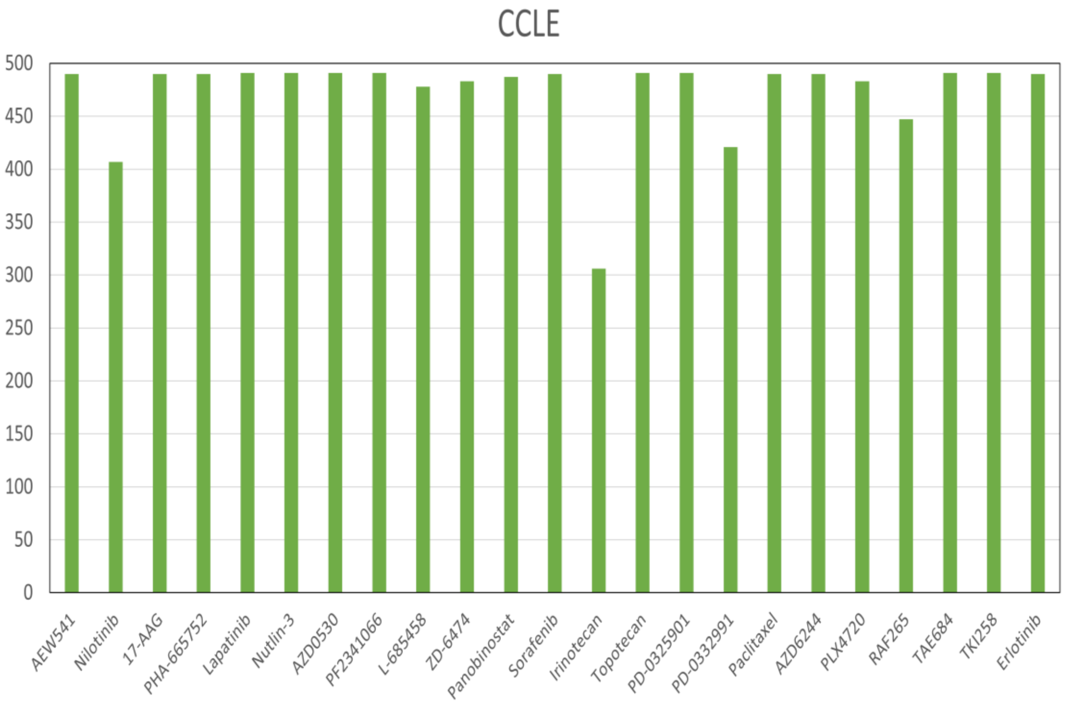


**Supplementary Table 1.** The ranked cell lines for each drug according to the predicted response values of WGRMF in GDSC. We applied WGRMF on GDSC dataset to predict the response values for those drug-cell line pairs without known

responses. The cell lines were ranked according to the predicted response values for each drug in GDSC, and the top 20 cell lines were selected for each drug. This prediction result is released for further experimental validation and research.
